# Worst-case scenario intubation of laryngeal granuloma: a case report

**DOI:** 10.1186/1756-0500-7-74

**Published:** 2014-02-03

**Authors:** Junko Nakahira, Toshiyuki Sawai, Sayuri Matsunami, Toshiaki Minami

**Affiliations:** 1 Department of Anesthesiology, Osaka Medical College, 2-7 Daigaku-machi, Takatsuki, Osaka, Japan

**Keywords:** Airway Scope®, Laryngeal granuloma, Vocal fold tumour

## Abstract

**Background:**

Intubation of patients with laryngeal granulomas on the vocal folds are sometimes difficult to manage because of potential airway obstruction. Laryngeal granulomas usually have flexible stalks where they attach to the vocal folds. We report a worst-case scenario of dislocation of the laryngeal granuloma during induction of anaesthesia.

**Case presentation:**

We present a case of laryngeal granulomas on the posterior vocal fold. A 20-year-old woman had an approximately 10-mm tumour in the laryngeal arytenoid region. Manual ventilation resulted in the tumour lodging in the subglottis and the inflated cuff of the intubation tube successfully returned it to its original position during tube withdrawal. Images were obtained using an Airway Scope® (Hoya-Pentax, Tokyo, Japan) and a video laryngoscope.

**Conclusion:**

In our case, the tumour was benign and relatively small in size; therefore, we did not select tracheotomy as an airway management strategy. The case had a granulomatous tumour arising from the posterior vocal folds on the right side, and the tumour was very flexible. To promptly gain control of the airway in such a case using direct laryngoscopy, thus avoiding tracheotomy, other strategies are suggested, such as bronchoscopic visualization with awake or semi-awake intubation.

## Background

Intubation of patients with laryngeal granulomas on the vocal folds are sometimes difficult to manage because airway obstruction can occur. Laryngeal granulomas usually have flexible stalks where they attach and compared with polyps or laryngeal cancer, which are tightly attached to the larynx, intubation in patients with laryngeal granulomas must be performed carefully to avoid disrupting the tumour [1,2]. Although there are several previous case reports of successful management of the difficult airway with laryngeal tumours, ours is the first report of dislocation of a laryngeal granuloma during treatment. We report a worst-case scenario of dislocation of a laryngeal granuloma during induction of anaesthesia. We used several imaging devices, including an Airway Scope® (Hoya-Pentax, Tokyo, Japan), a video laryngoscope.

## Case presentation

**Figure 1:**
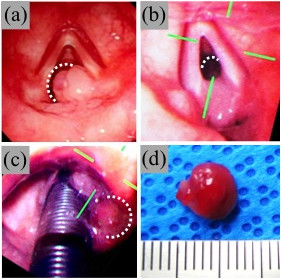
**Laryngoscopic images. (a)** Preoperative laryngeal endoscopy image. The dotted white line indicates the tumour. **(b)** Airway Scope® image after induction of general anaesthesia. Only the stalk of the tumour was visible, and the remaining section (dotted white line) was thought to be lodged in the subglottis. **(c)** Airway Scope® image after manoeuvring the intubation tube. The tumour (dotted white line) re-appeared at the supraglottic area. **(d)** An image of the approximately 8 mm diameter tumour.

**Figure 2:**
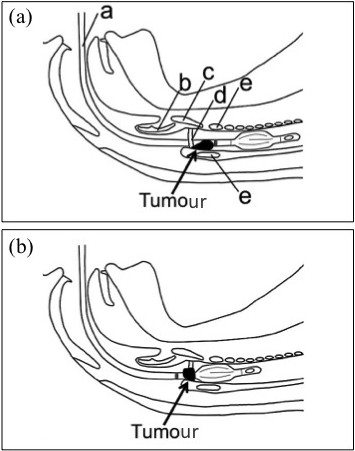
**Schematic demonstrating manoeuvring the tracheal tube. (a)** Drawing showing intubation to an insertion depth of 21 cm. **(b)** Drawing showing the endotracheal tube being slowly withdrawn to 16 cm. The tumour became visible at the supraglottis by manoeuvring the endotracheal tube. a: tracheal tube, b: epiglottis, c: thyroid cartilage, d: vocal fold, e: cricoid cartilage.

A 20-year-old woman, 157 cm tall, weighing 49 kg, complained of hoarseness after extraction of four wisdom teeth under general anaesthesia at another hospital 1 month earlier. She visited our hospital for consultation and treatment because the frequency and duration of the hoarseness had increased, causing intermittent, complete loss of vocalization. She had no symptoms of dyspnoea or discomfort in the larynx and no history of smoking, alcohol abuse, or other significant issues. She presented without trismus and was assessed as Mallampati Class I. Laryngeal endoscopy, performed at the otolaryngology department of our hospital, demonstrated a laryngeal tumour with a diameter of approximately 10 mm (Figure 1a). Tumour resection by laryngomicrosurgery was scheduled for the day after the outpatient consultation. Propofol 80 mg and remifentanil 0.33 μg/kg/min were administered intravenously for anaesthesia induction. Based on the size of the tumour, the possibility of complete tumour-induced tracheal and vocal cord obstruction was considered extremely low. Therefore, to suppress patient movement during intubation, 50 mg rocuronium bromide was administered after verifying that mask ventilation was possible, and positive pressure mask ventilation was performed for three minutes using 5% sevoflurane. During the first minutes of positive pressure ventilation, auscultation of the larynx revealed wheezing sounds. Using an Airway Scope® fitted with a single-use thin blade for adults (ITL-T, Pentax, Tokyo, Japan), the laryngeal tumour was found partially lodged in the glottis (Figure 1b). Only the neck of the tumour was visible on the vocal fold. Intubation was attempted by advancing a spiral tracheal tube with a 5.5 mm inner diameter and 7.5 mm outer diameter (Mallinckrodt Pharmaceuticals, St. Louis, MO, USA) from the right corner of the mouth using a stylet. The tumour was seen positioned partially between the tube and the trachea. The cuff was inflated with a cuff pressure of 30 cm H_2_O. Because it was not feasible to perform tumour resection using a laryngomicroscope under these conditions, otolaryngologists were consulted, and extraction of the tumour to the supraglottic area was attempted. To obtain an adequate visual field of the right vocal fold, the tracheal tube was first moved to the left side of the mouth, and laryngoscopy was performed using the Airway Scope® from the right side of the tracheal tube. The tube was slowly withdrawn along the left side of the mouth, 1 mm at a time, until the cuff was visible distal to the vocal fold, while maintaining cuff inflation. With this manoeuver, the tumour was successfully repositioned supraglottically (Figures 1c, 2a and b). Next, to prevent dislocation of the arytenoid cartilage by the repositioned cuff, the tracheal tube was slowly advanced 2 cm with the cuff deflated and fixed to the left of the mouth at a depth of 18 cm, while monitoring to ensure that the tumour did not re-invaginate. Surgical excision of the tumour was then performed. Intraoperative direct laryngoscopy showed that the tumour had a smooth surface with a wide neck, and its stalk adhered to the posterior part of the right vocal fold (Figure 1d). We saw no bleeding due to tumour involution by the tracheal tube. Postoperative histological examination led to a diagnosis of laryngeal granuloma. Laryngeal endoscopy performed the day after the procedure showed that although tissue protrusion remained at the posterior right vocal fold, no residual tumour was present and vocal tests demonstrated the absence of hoarseness.

## Discussion

The patient’s symptoms developed following general anaesthesia for dental extractions. Based on the clinical course of increasing frequency and duration of hoarseness, it was reasonable to presume that she had post-intubation granuloma. In this case, the location of the tumour changed after manual ventilation, becoming partially lodged in the glottis by the positive pressure ventilation. Therefore, ideally, awake or semi-awake intubation should have been performed. In cases with large or malignant tumours that can obstruct the glottis and trachea, tracheotomy can be performed, but the tumour was benign and relatively small in size in this case; therefore, we did not select tracheotomy as an airway management strategy. Also, tracheotomy is contraindicated in cases of laryngeal papilloma caused by human papillomavirus, which could not be ruled out preoperatively in this case [3].

Our case had granulomatous tumour arising from the posterior vocal folds on the right side, and the tumour was benign and very flexible. To promptly gain control of the airway in such a case using direct laryngoscopy, thus avoiding tracheotomy, other strategies are suggested. Awake or semi-awake intubation can be performed to prevent tumour disruption, and intubation can be performed by advancing the endotracheal tube along the opposite side from the tumour under bronchoscopic visualization. If the tracheal tube is advanced along the tumour side, the tumour becomes unobservable. Creative strategies, such as modifying the angle of the stylet, are necessary. In cases of dislocation and lodging of laryngeal tumours, the most appropriate device for visualization, such as a bronchoscope, should be prepared.

## Conclusions

We report a worst-case scenario of dislocation of a laryngeal granuloma during induction of anaesthesia. Advanced devices should be equipped and creative strategies for each case are needed.

## Consent

Written informed consent was obtained from the patient for publication of this case report and any accompanying images. A copy of the written consent is available for review by the Editor-in-Chief of this journal.

## Competing interests

The authors declare that they have no competing interests.

## Authors’ contributions

JN performed the anaesthesia and all authors discussed each case both preoperatively and postoperatively. JN and TS were major contributors to writing the manuscript, and SM and TM helped create the figures. All authors have read and approved the final manuscript.
